# Supplementary material for: SARS-CoV-2 infection of human lung epithelial cells induces TMPRSS-mediated acute fibrin deposition
Source: Nat Commun. 2023 Oct 11;14:6380. doi: 10.1038/s41467-023-42140-6 (PMC10567911; doi:10.1038/s41467-023-42140-6)
Supplement: Supplementary file 1 — Supplementary Information [file 41467_2023_42140_MOESM1_ESM.pdf]

SUPPLEMENTARY INFORMATION

SARS-CoV-2 infection of human lung epithelial cells induces TMPRSS-mediated acute fibrin deposition

Supplementary Figure 1

A

	C3146	C3189	C3263	C3267	R3151	R3188	R3232	R3248	H878	H902	H906
C3146	1	0.64	0.56	0.64	0.56	0.62	0.59	0.61	0.57	0.64	0.54
C3189		1	0.89	0.93	0.94	0.95	0.92	0.72	0.61	0.66	0.61
C3263			1	0.92	0.79	0.93	0.86	0.67	0.63	0.70	0.66
C3267				1	0.95	0.95	0.96	0.75	0.63	0.68	0.66
R3151					1	0.96	0.92	0.64	0.64	0.69	0.65
R3188						1	0.97	0.75	0.62	0.68	0.66
R3232							1	0.69	0.60	0.66	0.62
R3248								1	0.72	0.82	0.67
H878									1	0.67	0.70
H902										1	0.85
H906											1

B

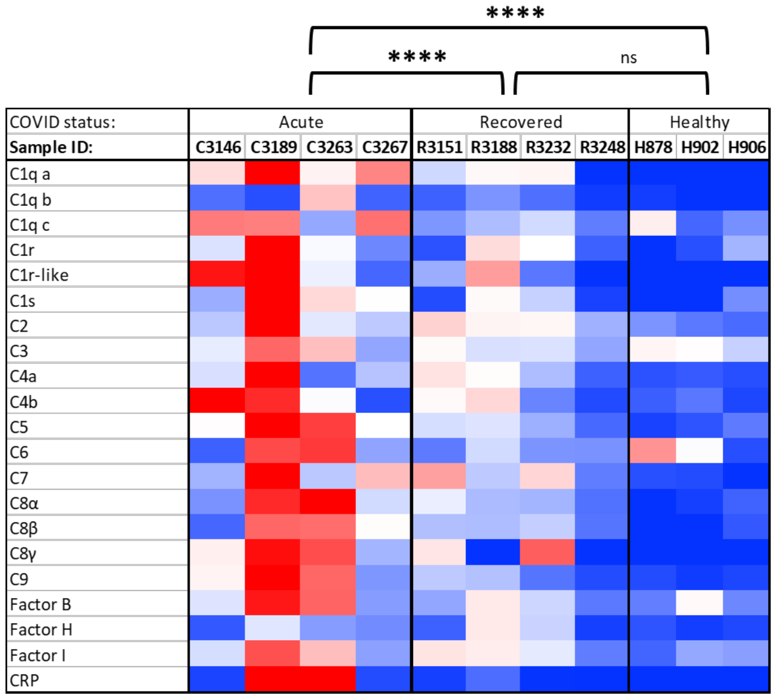

C

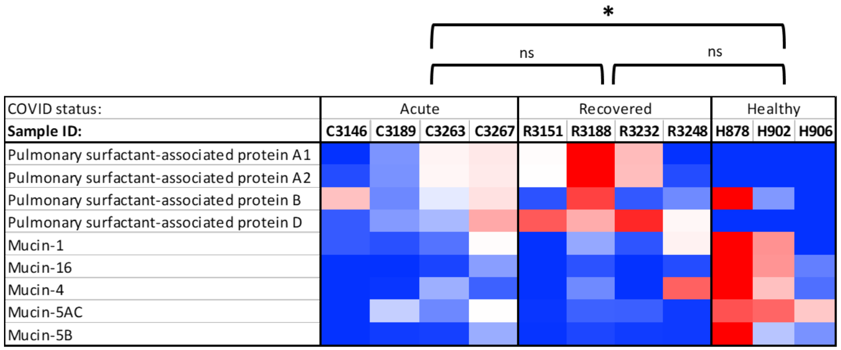

D

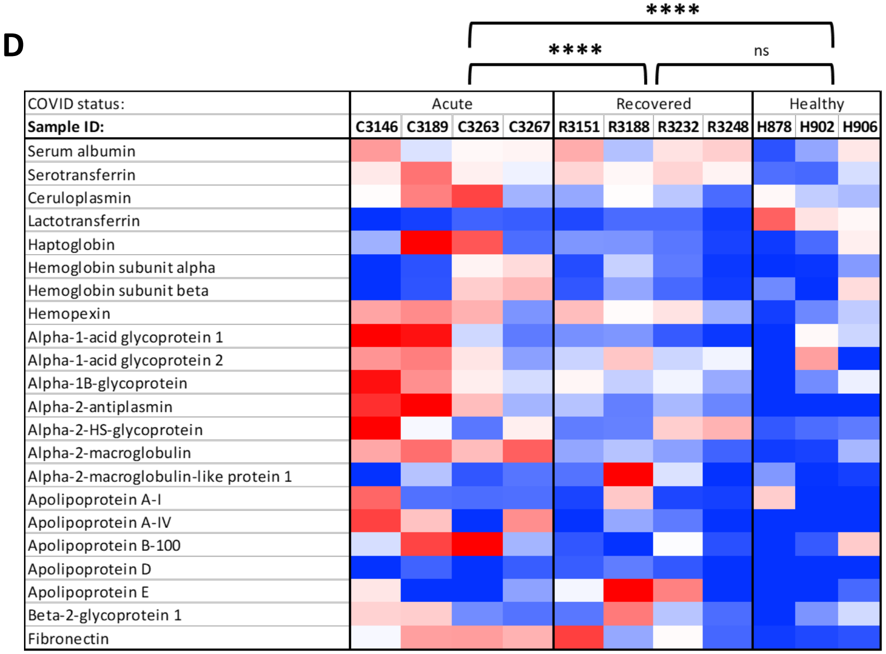

E

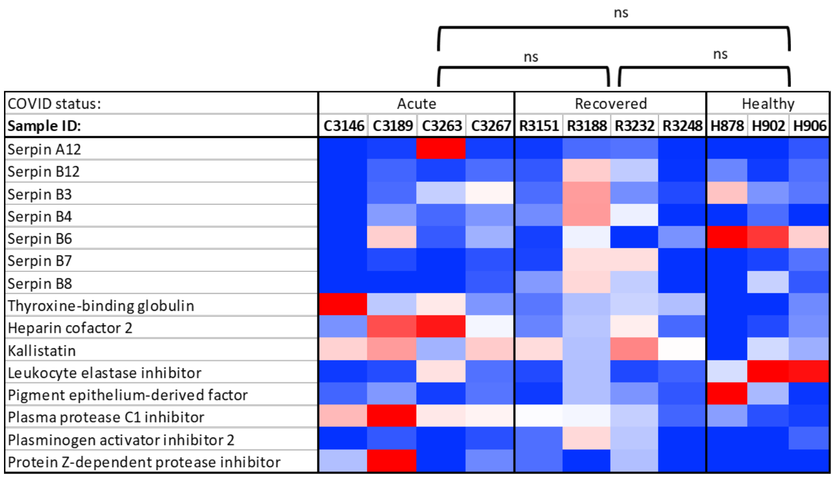

Supplementary Figure 1 Proteomic analyses of COVID and healthy BALF. A) Number of proteins identified and their overlaps among various BALF samples by mass spectrometry. B-E) Heatmap displaying differential abundance of mass spectrometry identified BALF proteins as part of complement pathway (B); pulmonary function (C); common serum proteins (D) and members of serpins (E). Samples labeled with C, R, and H denote acute- COVID, recovered-COVID and healthy BALF, respectively. The abundance of complement and common serum proteins but not Serpins are significantly increased. Statistics were calculated using 2-way ANOVA with p-values significant for column analysis, *p-value = 0.0126, **** < 0.0001.

Supplementary Figure 2

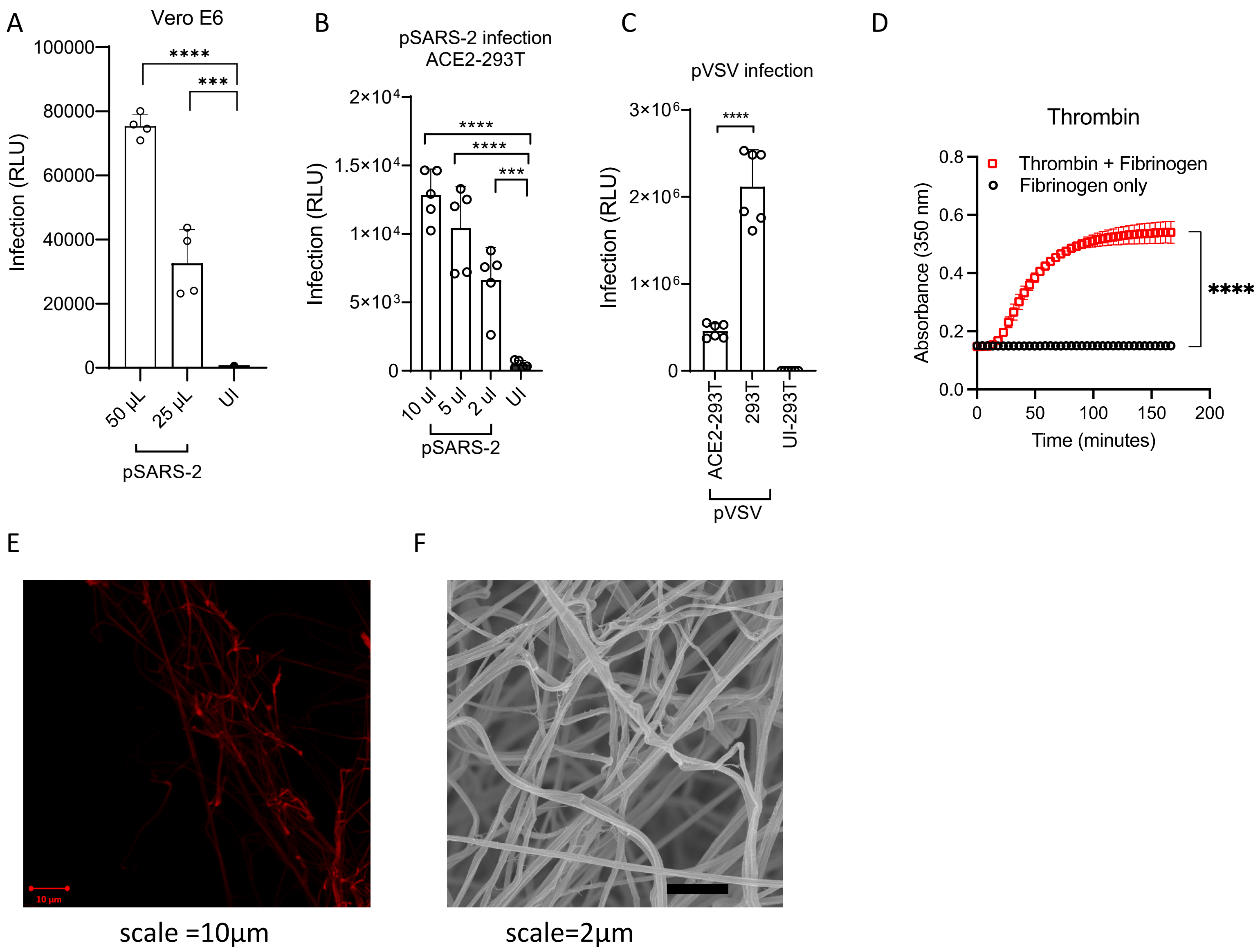

Supplementary Figure 2. SARS-CoV-2 pseudovirus infection and fibrin clotting.
A-C) SARS-CoV-2 dose-dependent infection of Vero E6 (A) and ACE2-293T (B) cells as well as VSV pseudoviruses infection of ACE2-293T and 293T cells (C). Cells were infected with pseudoviruses for 24 hours and lysed to measure luciferase activities. D) Thrombin catalyzed fibrin clotting. Thrombin was added to purified fibrinogen, and fibrin clot formation was measured by turbidity assay. OD was read with a plate reader at 350 nm. Data shows means ± SD. Statistics were performed using unpaired t tests with p-values * <0.05, *** <0.0005, **** < 0.0001. E-F) Confocal (E) and SEM (F) images of thrombin-catalyzed fibrin clotting. Thrombin was added to fluorescently labelled fibrinogen. Fibrin clot formation was visualized with confocal microscopy.

Supplementary Figure 3

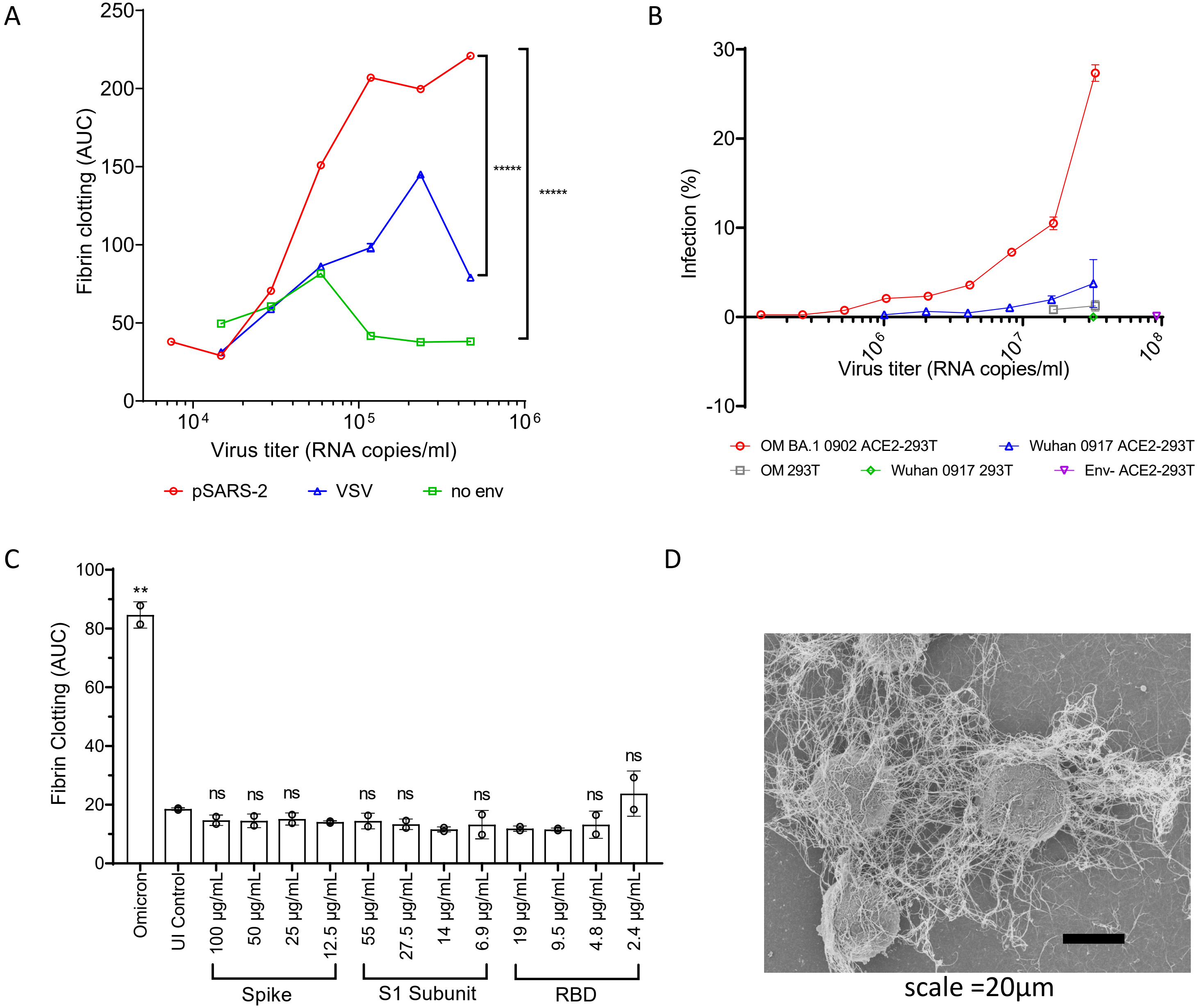

Supplementary Figure 3. Dose dependent SARS-CoV-2 pseudovirus infection. A) Viral dose dependent fibrin clotting from NHBE cells infected with SARS-CoV-2, VSV as well as envelope-null pseudoviruses presented as Area Under the Curve (AUC). B) Titration dose infection of ACE2-293T cells with omicron BA.1 (red), Wuhan (blue) or Env- (magenta) pseudoviruses. Their infection of 293T cells are used as further controls. The Env-null virus showed ~100x less infection compared to the omicron and Wuhan spike containing pSARS-2. C). Fibrin clot formation from NHBE cells treated with various concentrations of soluble Wuhan spike protein, S1 subunit or RBD domain, or in the presence of omicron pSARS-2 or mock (UI, untreated and uninfected) infections. Data are presented as mean values ± SD. Statistical analyese were performed between individual treatment and uninfected control using multiple unpaired t-test with p-values < 0.005 (**). The clotting levels of various recombinant spike, S1, or RBD treated samples are not significantly different from the uninfected/untreated control. D) SEM image of fibrin clots associated with SARS-CoV-2 pseudovirus infected NHBE cells. Scale bar=20 μm.

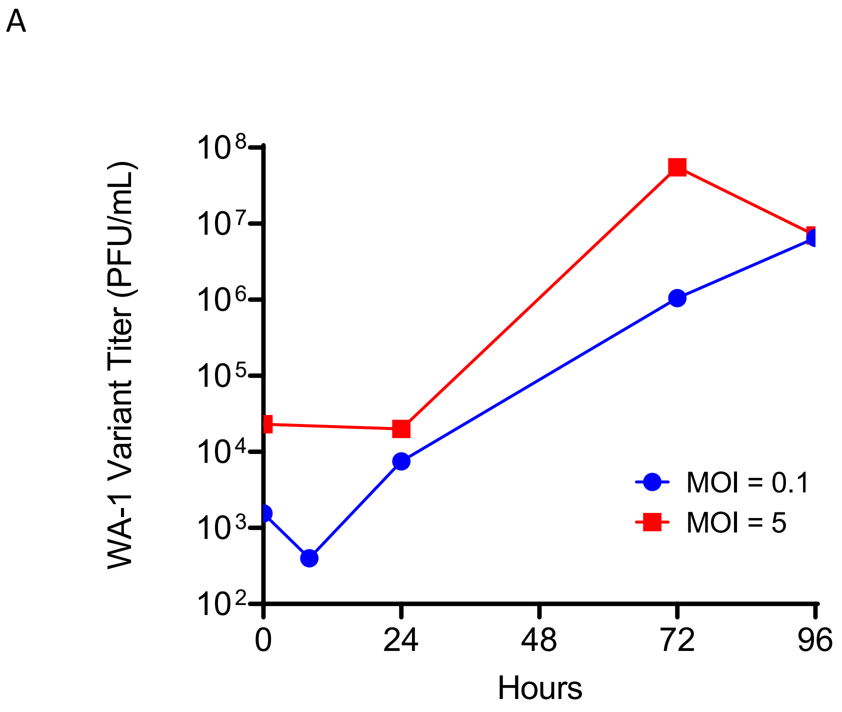

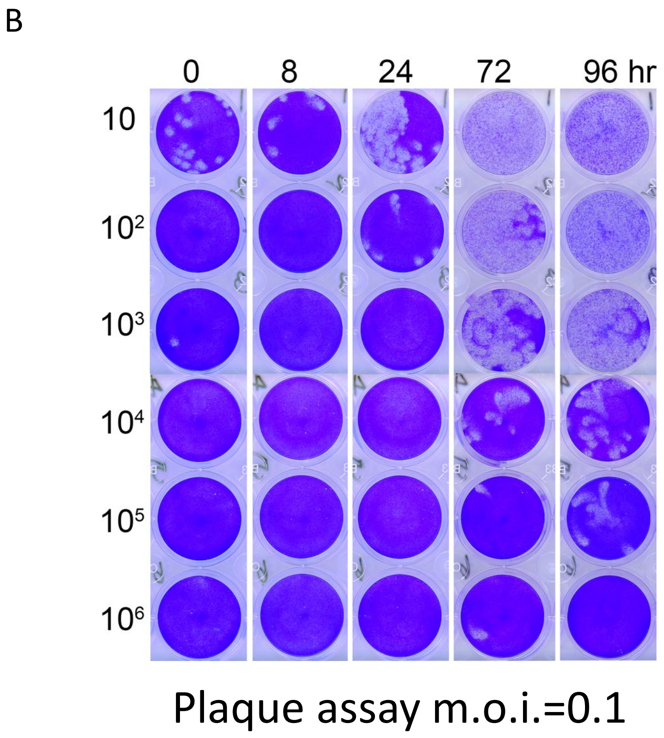

Supplementary Figure 4. WA-1 strain of SARS-CoV-2 infection of air-liquid interface cultured NHBE cells. A) The kinetics of viral titer expansion in infected NHBE cells. B) An example of plaque assay used to determine the viral titer at each time point.

Supplementary Figure 5

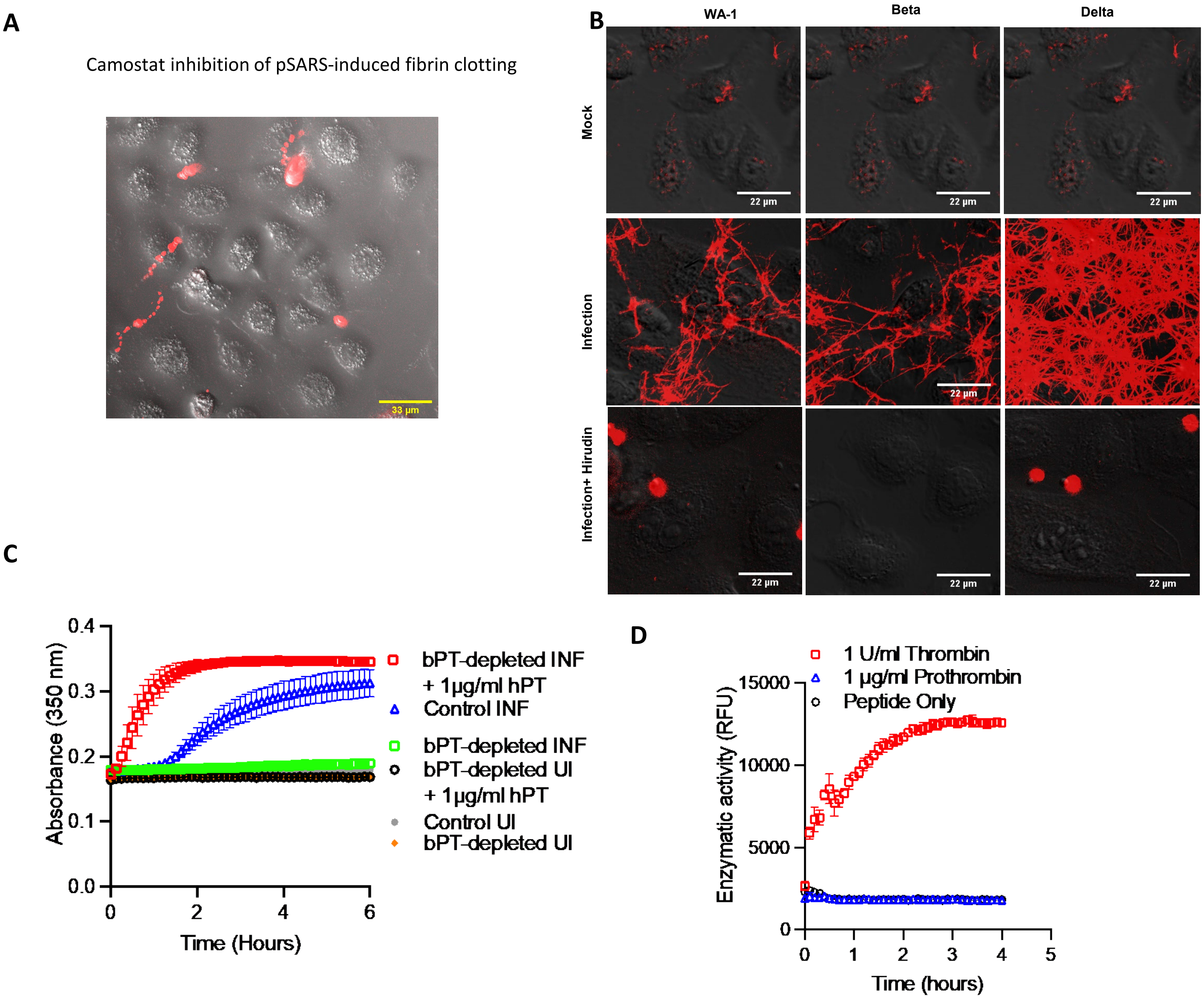

Supplementary Figure 5. Inhibition of SARS-CoV-2 infection-induced fibrin clot formation. A) Fibrin clot formation induced by Wuhan pSARS infected NHBE cells was suppressed by a serine protease inhibitor, camostat. B) Hirudin inhibited the fibrin clot formation by WA-1, beta, and delta strains of replication competent SARS-CoV-2 infection of NHBE cells. C) Fibrin clotting of SARS-CoV-2 infected (INF) or uninfected (UI) NHBE cells in the presence of normal (Control) or bovine prothrombin (bPT) depleted culture media with or without 1 μg/ml supplement of human prothrombin (hPT). D) Enzymatic cleavage of a fluorogenic peptide Dabcyl-SARGHRPLE(Edans), corresponding to the thrombin cleavage region of the N-terminal fibrinogen-β residues 42-49. The peptide cleavage indicates generation of activated thrombin as only the activated thrombin (red) but not prothrombin (blue) cleaved the peptide. The control reaction with only peptide but no enzymes is shown in green symbols. Data are presented as mean values ± SD.

Supplementary Figure 6

A

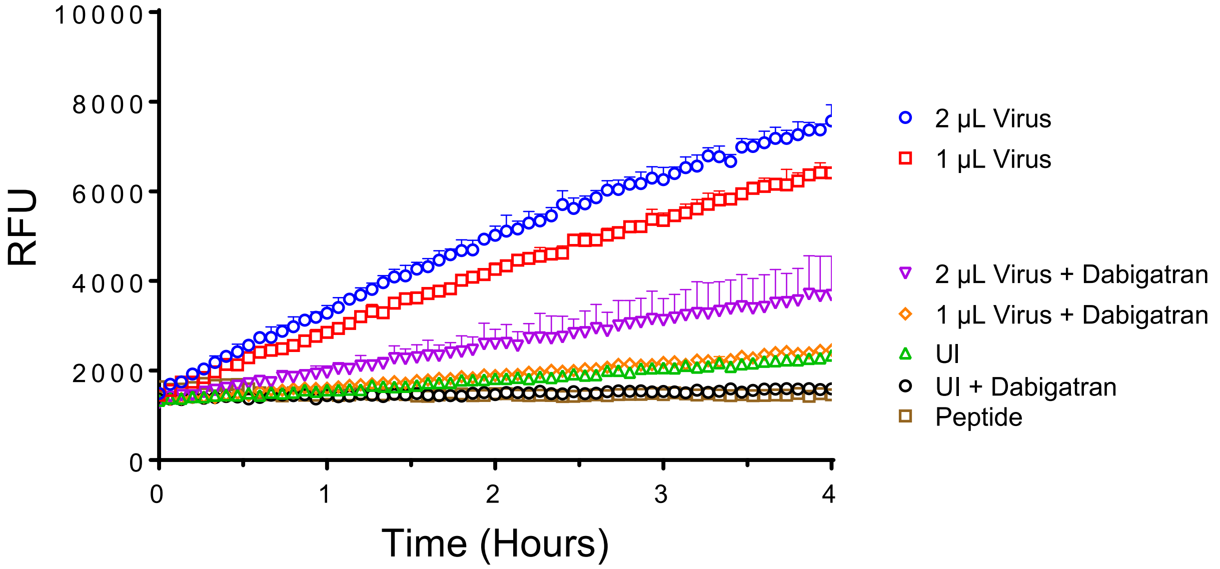

B

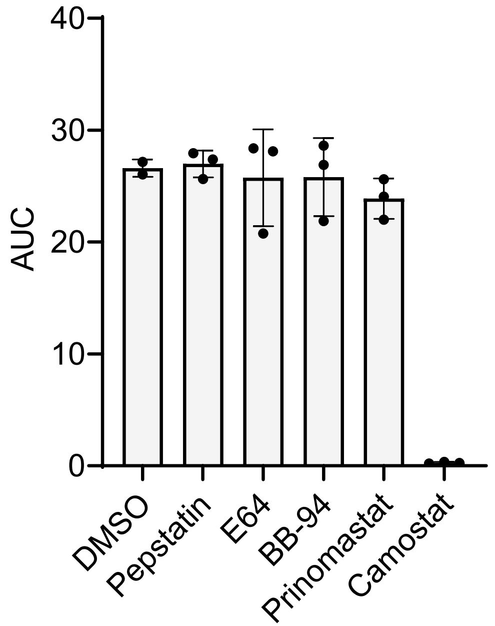

Supplementary Figure 6. Inhibition of infection-induced fibrin clotting by various inhibitors. A). Cleavage of fluorogenic fibrinogen-β peptide by SARS-CoV-2 infected or uninfected NHBE supernatants in the presence and absence of 10 μM thrombin inhibitor, dabigatran. B). Inhibition of the clotting step by Wuhan pSARS infected NHBE supernatants. As BB-94 reduced fibrin clot formation in Figure 6D, further experiment was performed to clarify if the inhibition by BB-94 was on the infection or fibrin clotting steps. In this figure, the fibrin clot formation was performed in the presence of various protease inhibitors. This figure differs from Figure 6D in that the inhibitors in this figure were added post infection during the fibrin clotting assay but not during the infection, whereas the inhibitors in Figure 6D were included during the infection. Thus, BB-94, an inhibitor for ADAM metalloproteinases, reduced fibrin clot formation only if it was added during the infection but not during clotting, supporting the shedding of transmembrane serine proteases is important in the infection-induced fibrin clot formation. Data are presented as mean values ± SD.

Supplementary Figure 7

A

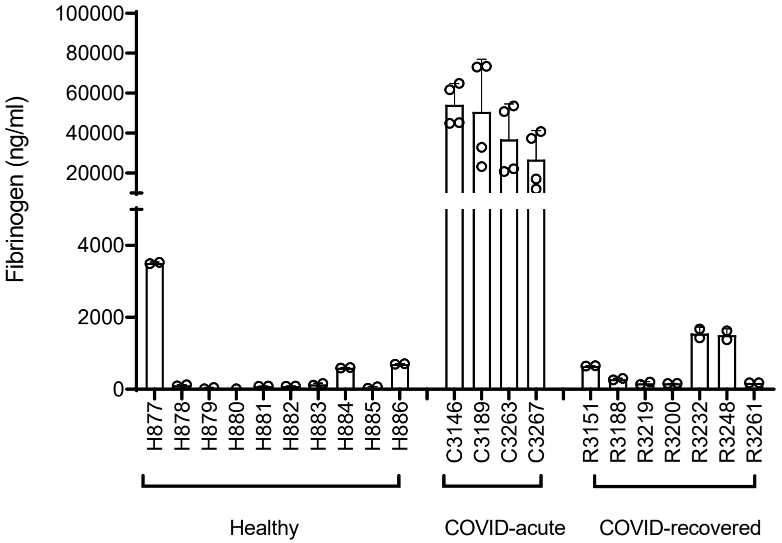

B

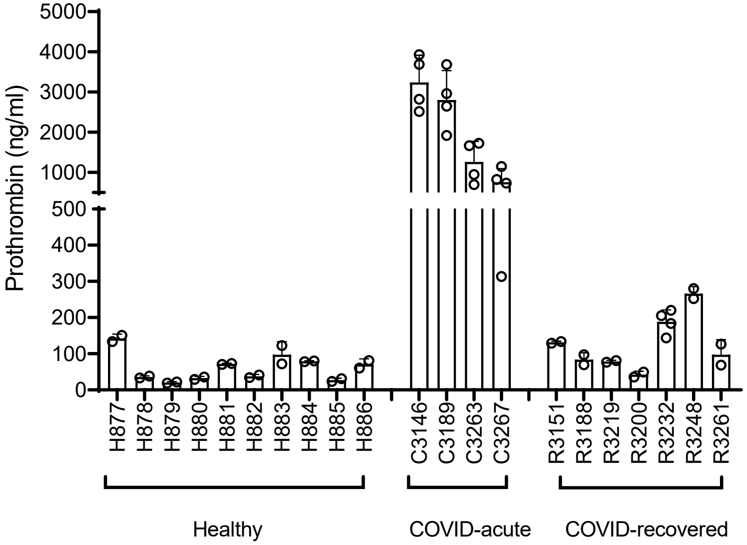

Supplementary Figure 7. Concentration of clotting factors in BALF. A-B) Fibrinogen (A) and prothrombin (B) concentrations in various healthy and COVID BALF as measured by ELISA. Data are presented as mean values ± SD.

Supplementary Figure 8

A

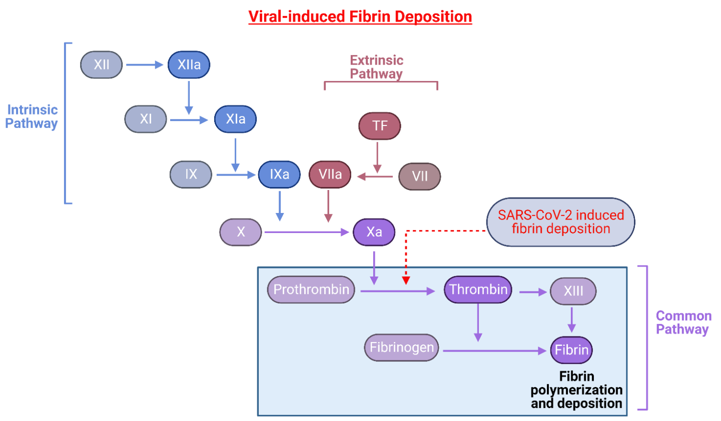

B

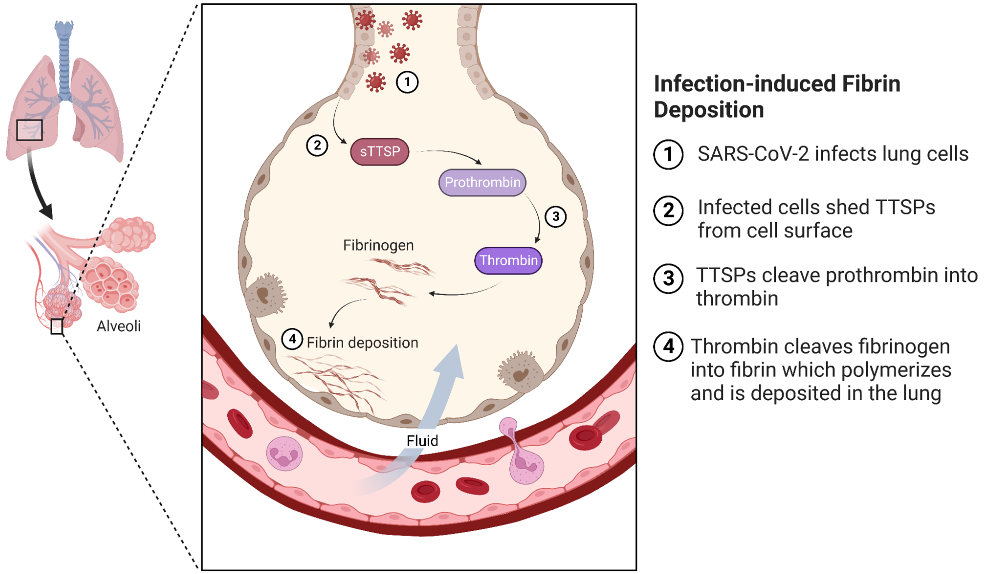

Supplementary Figure 8. A model for SARS-CoV-2 infection-induced fibrin deposition. A) The SARS-CoV-2 viral infection direct activates prothrombin for fibrin clot formation. The viral-induced fibrin clotting does not require classical coagulation factors. B) A model for SARS-CoV-2 infection induced fibrin deposition in lung. Both panel A and B are created with BioRender.com
