# Supplementary material for: SARS-CoV-2 infection of human lung epithelial cells induces TMPRSS-mediated acute fibrin deposition
Source: Nat Commun. 2023 Oct 11;14:6380. doi: 10.1038/s41467-023-42140-6 (PMC10567911; doi:10.1038/s41467-023-42140-6)
Supplement: Supplementary file 3 — Description of Additional Supplementary Files [file 41467_2023_42140_MOESM3_ESM.pdf]

Description of Additional Supplementary Files

Supplementary Data 1. A list of proteins in BALF identified by mass spectrometry.

Lists the proteins identified in various BALF samples with their normalized abundance. The BALF samples are identified as C3146, C3189, C3263, and C3267 from acute COVID, R3151, R3188, R3232, and R3248 from recovered COVID, and H878, H902, H906 from healthy donors, respectively. The list contains ~660 proteins that are grouped into the following categories: pulmonary proteins, common plasma proteins, coagulation factors, serine protease inhibitors (SERPIN), complement factors, immunoglobulins and other proteins. The corresponding proteomic data have been deposited to the ProteomeXchange Consortium via the PRIDE partner repository (http://www.ebi.ac.uk/pride) under identifier PXD045119.
